# Prevalence and microbiological characteristics of clinically infected foot-ulcers in patients with rheumatoid arthritis: a retrospective exploratory study

**DOI:** 10.1186/s13047-015-0099-0

**Published:** 2015-08-16

**Authors:** Pauline Fitzgerald, Heidi J. Siddle, Michael R. Backhouse, E. Andrea Nelson

**Affiliations:** Harrogate Hospital, Lancaster Park Road, Horrogate, HG2 7SX UK; Foot Health Department, Leeds Teaching Hospitals NHS Trust, Leeds, UK; Leeds Institute of Rheumatic and Musculoskeletal Medicine, University of Leeds, Leeds, UK; School of Healthcare, University of Leeds, Leeds, UK

**Keywords:** Infection, Foot ulcer, Rheumatoid arthritis, Wound swab

## Abstract

**Background:**

The prevalence of foot ulcers in patients with rheumatoid arthritis (RA) has been reported at almost 10 %. These foot ulcers often occur at multiple sites and are reoccurring, with the potential risk of infection increased due to RA diagnosis and disease modifying medications. The objective of this study was to estimate the prevalence of clinical infection in foot-ulcers of patients with RA; describe the microbiological characteristics and investigate risk factors.

**Methods:**

Retrospective clinical data was collected for all patients attending a rheumatology foot ulcer clinic between 1st May 2012 and 1st May 2013: wound swab data was collected from those with clinical infection.

**Results:**

Twenty-eight patients with RA and foot-ulcers were identified; eight of these patients had clinical infection and wound swabs taken (29 %).

Of these eight patients there were equal men and women, with median age 74 years, and average disease duration 22 years.

Cardiovascular disease/peripheral-vascular disease (CVD/PVD) were reported in six patients, diabetes in two patients.

Six patients were treated with disease-modifying anti-rheumatic drugs (DMARDs); three were on biologic medications and two on steroids.

Five wound swabs cultured skin flora, one staphylococcus aureus, one had no growth after culture; and one was rejected due to labelling error.

**Conclusion:**

Almost a third of people with RA and foot ulcers attending clinic over one year had clinical infection, however microbiological analysis failed to isolate pathogens in six of seven wound swabs. This may be due to inaccurate diagnosis of ulcer infection or to issues with sampling, collection, transport, analysis or reporting. There was insufficient data to relate risk of clinical infection with risk factors.

Further research is required to identify the most appropriate techniques for infection diagnosis, wound sampling and processing.

**Trial registration:**

Ethical approval was obtained from University of Leeds, Faculty of Medicine and Health (Reference number: SHREC/RP/349).

## Background

Between 50 and 93 % of patients with RA will have foot and ankle symptoms during the course of their disease; these have a marked impact on quality of life and mobility [1–3]. Persistent synovitis in the foot is associated with periarticular erosions and deformity throughout the foot [1] possibly leading to difficulty accommodating footwear and increasing plantar pressure [4]. Over time foot deformity and trauma from footwear can increase the risks of damage to surrounding skin, resulting in loss of skin integrity which may lead to foot-ulcers [5]. A foot-ulcer is defined as a skin defect including both the epidermis and the dermis, occurring in isolation on or below the midline of the malleoli and requiring ≥14 days to heal [6, 7].

The prevalence of foot-ulcers found in a self-report postal study of RA patients in Bradford, West Yorkshire was almost 10 %, however if patients with coexisting diabetes were excluded this would change to 3.13 % [5]. These foot-ulcers of patients with RA often occur at multiple sites and almost 50 % of patients suffer from recurrent ulcers [5, 7]. Although ulcers are usually small healing is slow, which increases the potential for infection [7]. Due to the increased risk of developing skin and soft tissue infections in patients with RA [8] minimising potential risk should be a priority for practitioners. Being able to identify an infected foot-ulcer is essential to provide appropriate care, including decisions on continuation of immunosuppressive and immune-modulating medications. Clinical diagnosis of infection is determined through signs and symptoms and reliant on the experience of individual practitioners [9], although the validity of such a tool has yet to be proven in RA patients. Wound infection is defined as multiple bacteria present which can cause damage to wound tissue and delay healing [10]. Following a diagnosis of clinical infection, identification of micro-organisms using a swab, or tissue sample, allows for targeted treatment and appropriate use of antibiotics, which is essential to reduce the incidence of bacterial resistance [11]. In the authors experience microbiology reports typically take several days to reach the clinician so selection of first line, empirical, antibiotic therapy is based on prior knowledge of likely infective organisms in the specific clinical population.

Despite the known burden of foot ulcers in RA [6] there remain large gaps in knowledge of the sequelae and treatment of this population. The prevalence and microbiological characteristics of infected foot-ulcers have not previously been identified so there is a lack of information to guide first line antibiotic selection in patients with RA.

The primary aim of the study was to identify the prevalence of clinically infected foot-ulcers in patients diagnosed with RA, who attended a foot ulcer clinic of a large teaching hospital and subsequently identify and quantify the types of microorganisms found in those ulcers.

The secondary aim of the study was to identify relationships between the types of microorganisms identified in the ulcer and previously identified risk factors for infection in patients with RA.

## Methods

This descriptive epidemiological study identified the number of RA patients with clinically infected foot ulcers. The microorganisms were identified and quantified through microscopy culture results.

**Fig. 1:**
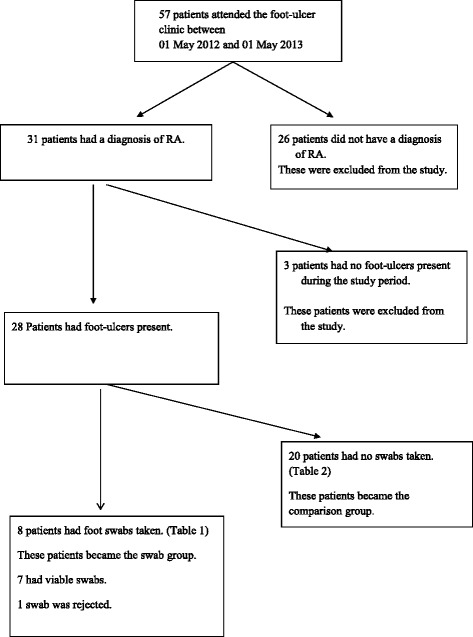
Flow Chart. Flow Chart to identify the eight patients with RA included in the analysis

Retrospective data was obtained from clinic notes and electronic records of all patients attending the weekly rheumatology foot-ulcer clinic in a large NHS teaching hospital Trust between 1st May 2012 and 1st May 2013; this determined the population size [Fig. 1].

All patients with a consultant diagnosis of RA who presented with an active foot-ulcer and had a wound swab taken between 1st May 2012 and 1st May 2013 became the main focus of the study [swab group]. If multiple wound swabs were taken, the first taken in the study period was identified as the index swab. All microbiology reports were collected from 12 months prior to the index swab date and the three months after. This 15 month review of microbiology reports allowed the researchers to capture the reoccurring nature of foot-ulcers and infection. Due to time constraints the majority of data was retrospective, pre-dating the index swab. Wound swabs taken above the midline of the malleoli were excluded.

The patients who had RA and foot ulcers but no swabs taken became the comparison group and had minimal data collected from their first clinic attendance during the study period. This enabled comparisons of age, gender and disease duration to be made between the two groups.

The dependent variables investigated were the microbiological characteristics identified from swab results obtained from the foot ulcers of the RA patients in the study.

The independent variables included the possible contributory factors, other than RA, as the causation of the foot-ulcer infection, these included: Demographic data, current medications, disease activity scores (DAS 28) [12], comorbidities of diabetes, connective tissue disease, cardiovascular / peripheral vascular disease (these terms include myocardial infusion [MI] Cerebrovascular accidents [CVA] Transient ischaemic attack [TIA].) and smoking habits.

Gender details were collected to see if the 3:1 (Female: Male) incidence of developing RA and foot-ulcers [7, 13] are reflected in the numbers developing infection.

Immunosuppressant and steroid medications used to treat RA, can increase the risk of infection by their very action of dampening the autoimmune response. With anti-TNF therapies being linked to an increase in skin and soft tissue infections [14–16].

RA disease activity (reflected in the DAS 28 score) [12] has been linked to infection with an increased risk of infection seen in patients with higher DAS 28 scores [17, 18].

Diabetes is known to be a major risk factor for the development of foot ulcers [19, 20] and this together with delayed healing, increased infection risk, neuropathy and peripheral vascular disease all contribute to a higher risk of infection in the foot ulcers [21–23].

Connective tissue diseases (CTD) are often associated with self-reported foot problems [24] particularly when conditions affect the circulation and/or peripheral sensation [25].

Cardiovascular/Peripheral-vascular Disease (CVD/PVD) can cause decreased blood flow to the foot ulcer resulting in depleted oxygen supply so creating hypoxia within the wound. Although hypoxia can stimulate wound healing and angiogenesis initially, sustained hypoxia prevents the supply of oxygen and nutrients needed for wound healing [26, 27].

Smoking cigarettes has been shown to delay wound healing [28, 29] and diabetic patients with foot ulcers who smoke are more likely to require amputation than non-smokers [30]. In RA patients smoking cigarettes has been linked with increased disease activity [31–33] and the development of rheumatoid nodules [34].

Descriptive statistical analysis of individual variables allowed central tendencies to be displayed.

Ethical approval was obtained from University of Leeds, Faculty of Medicine and Health (Reference number: SHREC/RP/349).

## Results

Fifty-seven patients attended the rheumatology foot-ulcer clinic during the study period, 31 of whom had a diagnosis of RA. Twenty eight of the 31 patients with RA had foot-ulcers and eight of those patients had wound swabs taken during the study period (Fig. 1). Therefore 28.5 % were considered to have clinical infection in their foot ulcers. One swab was rejected by the microbiology laboratory due to incomplete labelling.

Table 1 displays demographic data, current medications, DAS 28 and comorbidities for the eight patients.

**Table 1:** Patients diagnosed with RA, had foot ulcers present in the study period and had swabs taken. [Swab group]

Study Number	Age^a^ in years	Gender	Disease Duration^a^in years	Diabetes	CTD	CVD /PVD	Smoker Y/N	DAS:28 [within 4 weeks of index swab]	Standard DMARD therapy	Anti – TNF therapy	Other biologic therapy	Steroid therapy
1	82	F	N/A	N	N	Y	Y current	N/A	N	N	N	N
2	67	M	28	N	N	Y	N	N/A	Y	N	Y	Y
3	78	F	17	N	N	N	N	N/A	Y	N	N	N
4	85	M	26	Y	N	Y	N	N/A	N	N	N	N
5	67	M	15	N	N	Y	N previous	N/A	Y	N	N	Y
6	72	F	30	N	N	N	N previous	N/A	Y	N	Y	N
7	71	M	25	Y	N	Y	N previous	N/A	Y	N	N	N
8	76	F	13	N	N	Y	Y current	N/A	Y	N	Y	N
	Mean: 74.75	F = 4	Mean: 22	Y = 2	Y = 0	Y = 6	N = 6	No scores in notes.	N = 2	N = 8	N = 5	N = 6
	Median:74	M = 4	Median:25	N = 6	N = 8	N = 2	Y = 2		Y = 6	Y = 0	Y = 3	Y = 2

*CTD* connective tissue disease, *CVD/PVD* cardiovascular / peripheral vascular disease, *N/A* indicates missing data; *Y* yes, *N* no; *F* female, *M* male

^a^relates to index swab date

DAS 28 was not documented within 4 weeks of the index swab date for any of the eight patients (Table 1).

The characteristics of the twenty patients with RA and foot-ulcer/s who had no wound swabs taken between 1st May 2012 and 1st May 2013 (comparison group) are in Table 2.

**Table 2:** Comparison Group: RA patients with foot-ulcer but no swabs taken during study period or in the year before first attendance at the foot ulcer clinic

Age^a^ in years	Gender	Disease Duration^a^ in years
84	F	14
71	F	22
80	F	7
77	F	8
61	F	51
84	M	27
79	M	10
87	F	27
81	F	49
74	F	15
78	F	N/A
67	M	18
82	F	62
44	M	1
73	M	32
72	F	20
75	F	21
73	M	36
73	F	4
50	F	20
Mean: 73.25	F = 14	Mean: 23.3
Median:74	M = 6	Median: 20

*N/A* indicates missing data, *F* female, *M* male

^a^Indicates at first clinic attendance during study period

The microbiological profile (Table 3) was obtained from the swabs of the eight study patients. Seven of the patients had swabs taken from the toes and one was from the foot. The microbiology report provided the microscopy culture information (Table 3) for seven viable wound swabs. The swab results reported skin flora in five patients, one in conjunction with enteric flora (one toe swab) and three in conjunction with anaerobes. Staphylococcus aureus was isolated in one patient. No growth was reported in one swab.

**Table 3:** Microbiology Information collected from the patients with RA, had foot ulcers present in the study period and had swabs taken [swab group]

Study No.	Location of Ulcer	Clinical Picture	Microscopy Culture result	Sensitivity detected	Treatments/antibiotics used
1	Right 2^nd^ toe	Thick yellow pus type exudate coming from original ulcer and on exit wound of lateral border of apex. Same toe. Unable to probe as very painful.	Skin flora ++	Non specified	Co-Amoxiclav.
					10 weeks prescribed but only completed 3 weeks due to side effects.
			Swabs from before the index swab:		
			41 days before: scanty skin flora		
			39 days before: skin flora +		
		X-ray 3 months before index swab showed osteomyelitis.			
					Antibiotics before index swab:
		Bone removed 7 days after index swab for culture.			
					39 days before: Flucloxacillin
					41 days before: nil antibiotics
2	Left medial malleolus.	Granular spreading infection – lot of exudate and spreading to deep tissue – probing more than 10 mm. X-ray taken.	Swab rejected from microbiology as no location/no Consultant specified.	N/A	Flucloxacillin
		15 days before index swab: wound clean, a granular base with no evidence of infection. 4 mm x 4 mm.			
3	Plantar aspect of right 1^st^ toe, IP joint.	Index swab taken at start of ulcer.	Mixed skin and enteric flora +++	Non specified	Clarithromycin
		Ulcer dressed by district nurse before referral to podiatry 107 days later.			
4	Right 3^rd^ plantar, MTP joint.	Foot ulcer swab.	Skin flora ++ with anaerobe ++	Non specified	Flucloxacillin
5	Left 4^th^ IP joint.	Probe depth to bone. No clinical signs of infection.	Skin flora + with anaerobe +	Metronidazole	None listed.
6	Left 2^nd^ and 3^rd^ toes.	Ulcer *x*2 middle toes left foot.	Staphylococcus aureus +++.	Flucloxacillin sensitive	Flucloxacillin
			Documented as: May represent colonisation only. Suggest treat only if current clinical evidence of infection.	Penicillin Resistant	
				Clarith/Eryth Sensitive.	
7	Plantar lesion right hallux. 1^st^ MTP joint.	Deep ulcer under right hallux. Probing to bone. Aspirated bursa fluid sent to microbiology.	Gram stain: no organisms seen	Non specified	None
			Gram WBC: none seen		
			These samples are not routinely examined for crystals.		
			Culture: no growth		
8	Left big toe.	Infected rheumatoid nodule.	Skin flora ++ with anaerobe ++	Non specified	None stated
			May represent colonisation only.		
			Skin flora + with Anaerobe ++		
		14 days after index swab: RA infected Left big toe		Sensitive to Metronidazole	Flucloxacillin

The patient study numbers correlate with those in Table 1

*MTP* metatarsophalangeal, *IP* interphalangeal, *N/A* not applicable

Sensitivities identified by microbiology are reported in Table 3.

Only one patient was treated with the antibiotics identified in the microbiology report (Table 3 study no.6). Four patients were treated with antibiotics not specified as sensitive.

## Discussion

This is the first study to describe the prevalence of clinical infection in RA foot-ulcers and the microbiological characteristics of infections.

Heavy growth of one/mixed bacteria can disrupt healing and cause damage to wound tissue, typically seen in wound infections [10]. Only one of the seven swab results demonstrated this trait. This may be because despite clinical appearance there was no infection, or that sampling failed to collect and grow live organisms. Swabbing technique, the ability of the micro-organisms to survive in transit to the laboratory and the procedures within the laboratories [35, 36] all contribute towards the performance of swabbing as a way to identify organisms within a wound.

The skin is naturally colonised by many microorganisms, most of which are harmless to the host [37]. Normal skin flora varies around the body dependent on moisture levels, body temperature and concentration of skin surface lipids [37]. Skin areas with partial occlusion like toe webs, axilla and perineum often harbour more microorganisms than less occluded areas like the legs, arms and trunk [37]. Staphylococcus epidermidis is a major skin inhabitant often representing 90 % of resident aerobic flora [9]. Staphylococcus aureus is common, being present in 10 to 40 % of the adult population [37]. Other microorganisms such as micrococci, diptheroids, peptostreptococcus (anaerobic) and gram-negative bacilli also contribute towards skin flora [37].

Local practice, after clinical diagnosis of infection, was to collect a wound swab to identify microbiology causing the infection, therefore (as data collection was retrospective) the taking of a wound swab was interpreted as clinical infection, for the purpose of this study. Eight of the 28 patients (28.5 %) with RA foot-ulcers had swabs taken, presumably because the clinician diagnosed infection, this percentage of infection was lower than the authors expected, but comparable to the incidence of infection seen in diabetic foot ulcers (DFU) in other studies [38]. The potential prevalence of infection was possibly lower, as the microbiology results suggest inaccurate diagnosis of infection. Further study is clearly needed to describe the micro-organisms found in infected foot-ulcers in RA, to guide empiric therapy.

Ability of wound swabs to characterise the infection in DFU has been questioned in the literature [35, 36]. Studies have suggested that deep tissue biopsy may be more sensitive at detecting organisms and guiding subsequent antibiotic therapy [35, 39]. It has also been suggested that bacterial cultures inherently amplify the bacteria that grow easily and are unable to fully identify the diversity of all microorganisms that may be present in a wound [36]. Recently, the molecular method of real-time polymerase chain reaction (PCR) has been proposed for the identification of microorganisms with studies reporting greatly increased sensitivity when compared to traditional cultures [40]. Rhoads et al. [40] hypothesise that clinical outcomes for patients with chronic wounds could improve if this was used to identify bacteria but this is untested. Commonly cited indicators of infection in DFU include increasing pain, erythema, oedema, heat and purulent exudate, if used in combination they can provide a moderately reliable predictive tool [38]. A simple classification system of DFU infection combined with a vascular assessment has been shown to assist clinicians to diagnose and manage DFU infection care [41]. The validity of these indicators in an immunosuppressed population of patients with RA remains unclear.

Although there are guidelines for screening the feet of RA patients, which includes foot ulcers [42], there are no standardised guidelines or classification systems for diagnosing infection in the foot-ulcers of patients with RA; those used in chronic wounds [9] are not validated in this patient population. Consequently individual clinician’s decisions are reliant on experience and the evidence that supports diagnosing infection in other patient groups, like DFU [38].

The clinicians in this area may have high sensitivity and low specificity at identifying infection because of the high risk of, and consequences of, infection during immunosuppressive therapy, such as DMARDs, steroids and biologic therapies [43, 44]. With anti-TNF therapies being linked to an increase in skin and soft tissue infections [14–16]. DMARDs are recommended for RA patients, providing relief from symptoms and prevention of disease progression. The documented evidence, informing practitioners on safe use, recommends DMARDs are suspended in the face of infection [42, 45]. Six of the eight study patients were receiving these and 3 were receiving biologic therapies. If neutropenia develops as a result of these therapies then the risk from normal skin flora contributing towards the development of cellulitis where there is a break in the skin, or ulceration is increased. The risk of infection therefore may have led to swabbing to rule out infection, the swab may have been taken to reassure the clinician. However, clinical notes were not detailed enough to inform the research team of the clinicians reasoning behind swab collections.

The patients characteristics support the evidence that increasing age and disease duration impact the risk of developing foot-ulcers: However whether these also increase the risk of developing infection requires further study. Due to increasing age and decreasing dexterity, typical for these patients, personal hygiene ability requires consideration when evaluating risk.

Comorbidities of CVD/PVD are evident in a large proportion of the study patients, supporting previous research of an increased risk factor: However of the two diabetic patients’ only one cultured skin flora with anaerobes [37].

Limitations of the study include incomplete and missing data, as original data (patients’ notes) were collected during routine hospital appointments [46]. Furthermore a cross-sectional study design does not allow for longer term evaluation of the consequences of colonised skin flora in foot-ulcers of the study patients. The small population and single study site also limits reliability and generalisability of the study. The retrospective data collection used in the study meant the clinicians’ rationale for taking a swab was not available to the authors. Consequently this limits the interpretation of the primary aim of the study, to identify the prevalence of clinical infection in foot ulcers of RA patients.

## Conclusion

Almost 30 % of patients with foot ulcers and RA had swabs taken for wound infection (defined as clinical infection). The majority of microbiology results did not concur with the clinicians’ diagnosis of infection. The study was unable to conclude possible relationships between the types of microorganisms identified in the foot ulcers and previously identified risk factors for infection in this patient group.

The rational for clinical judgements and decisions was not available through retrospective data-collection, this needs further investigation to understand.

The process of collecting specimens for identification of colonised bacteria also needs further research to establish best practice and reliable methods.

However clinician diagnosed infection and cultured evidence of infection are two different concepts and clearer guidelines would inform the practitioner of when it is safe to continue therapy and when it should be with-held, so improving patient care.

More research is needed to investigate actual infection rates of foot-ulcers in RA patients, providing practice guidelines and standardisation of care.
